# Extracorporeal liver support systems in patients with acute‐on‐chronic liver failure: An updated systematic review and meta‐analysis

**DOI:** 10.1111/aor.14915

**Published:** 2024-11-22

**Authors:** Haiyu Liu, Zhibo Yang, Qiong Luo, Jianhui Lin

**Affiliations:** ^1^ Department of Pulmonary and Critical Care Medicine Fujian Medical University Union Hospital Fuzhou China; ^2^ Department of Clinical Medicine Clinical College of Anhui Medical University Hefei China; ^3^ Department of Oncology Medicine Mengchao Hepatobiliary Hospital of Fujian Medical University Fuzhou China; ^4^ Artificial Liver Center Mengchao Hepatobiliary Hospital of Fujian Medical University Fuzhou China; ^5^ Department of Liver Diseases Mengchao Hepatobiliary Hospital of Fujian Medical University Fuzhou China

**Keywords:** acute‐on‐chronic liver failure, extracorporeal liver support system, hepatic encephalopathy, hepatorenal syndrome, mortality, spontaneous peritonitis

## Abstract

**Background:**

The utilization of extracorporeal liver support systems is increasingly prevalent for the management of acute‐on‐chronic liver failure in clinical settings. Yet, the efficacy of these interventions in terms of tangible clinical benefits for patients remains a matter of debate, underscoring the need for meta‐analysis.

**Methods:**

An updated meta‐analysis was performed to elucidate the relationship between the application of extracorporeal liver support versus standard pharmacological treatment and the prognostic endpoints of patient survival, specifically assessing 1‐month and 3‐month mortality rates, as well as the incidence of complications such as hepatic encephalopathy, spontaneous bacterial peritonitis, and hepatorenal syndrome. Literature were searched via PubMed, EMBASE, and Web of Science.

**Results:**

The meta‐analysis revealed the following: the odds ratio for 1‐month mortality was 0.63 (95% confidence interval [CIs]: 0.51–0.76), for 3‐month mortality was 0.70 (95% CI: 0.61–0.81), for hepatic encephalopathy was 0.81 (95% CI: 0.67–0.97), for spontaneous bacterial peritonitis was 0.66 (95% CI: 0.44–0.99), and for hepatorenal syndrome was 0.68 (95% CI: 0.51–0.92). These results suggest that patients with acute‐on‐chronic liver failure undergoing extracorporeal liver support system therapy have significantly better survival rates and lower complication incidences compared to those receiving conventional drug therapy. Further subgroup analysis indicated that patients with lower model for end‐stage liver disease (MELD) scores and reduced total bilirubin (Tbil) levels demonstrated greater benefits from extracorporeal hepatic support.

**Conclusion:**

This study establishes that in the management of acute‐on‐chronic liver failure, extracorporeal liver support systems confer a survival advantage and reduce complications relative to standard pharmacotherapy.

## Graphical abstract

The meta‐analysis showed the following: 1‐month mortality odds ratio was 0.63 (95% CI: 0.51–0.76), 3‐month mortality was 0.70 (95% CI: 0.61–0.81), hepatic encephalopathy was 0.81 (95% CI: 0.67–0.97), spontaneous bacterial peritonitis was 0.66 (95% CI: 0.44–0.99), and hepatorenal syndrome was 0.68 (95% CI: 0.51–0.92). In conclusion, extracorporeal liver support systems improve survival and reduce complications compared to standard pharmacotherapy in acute‐on‐chronic liver failure management.

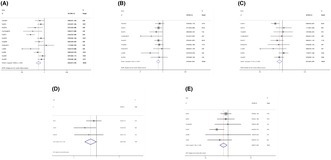

## 1 INTRODUCTION

Acute‐on‐chronic liver failure (ACLF) is a syndrome characterized by the sudden deterioration of hepatic function in individuals with previously stable chronic liver disease, precipitated by various acute insults.^1^ The syndrome manifests with a spectrum of severe clinical features, including extreme fatigue; pronounced gastrointestinal symptoms such as abdominal pain, distension, nausea, anorexia, and vomiting; jaundice with progressive intensification of skin and mucosal pigmentation; darkening of urine; and profound coagulopathy, evidenced by skin and mucous membrane hemorrhages, epistaxis, gingival bleeding, gastrointestinal hemorrhage, and hematuria. Low‐grade fever and other complications may arise, presenting with clinical features that vary according to the underlying liver failure subclassification. Temporally, ACLF is categorized into two types based on the progression to hepatic failure syndrome: acute‐on‐chronic liver failure, within two weeks of onset, and subacute‐on‐chronic liver failure, beyond this period. Both categories are encompassed by the term ACLF. The rapid progression of ACLF frequently results in adverse clinical events, significantly impairing patient prognosis and quality of life.

Extracorporeal liver support systems (ELSS), alternatively known as non‐bioartificial liver support systems or artificial liver support systems (ALSS), represent a critical advancement in therapeutic interventions for liver failure. These systems serve as a temporary surrogate for hepatic function by employing extracorporeal devices—either physicochemical or biological—to detoxify the patient's blood. By removing toxins and compensating for the liver's physiological functions, ELSS facilitates hepatic regeneration, bridging patients either to spontaneous liver recovery or to liver transplantation.^2^

This study addresses a critical gap in the existing literature regarding the efficacy of ELSS in patients with ACLF. Despite the potential of ELSS to extend survival and mitigate adverse clinical events, there remains a paucity of high‐quality evidence delineating its precise role and impact on mortality and morbidity, particularly in comparison to standard pharmacological therapies. This meta‐analysis aims to rigorously evaluate the hypothesis that ELSS confers a prognostic advantage in ACLF.

To achieve this objective, we will systematically review and synthesize data from studies comparing outcomes of ACLF patients treated with ELSS versus standard pharmacologic interventions. We will concentrate on quantifying the impact on prognostic endpoints, specifically focusing on patient survival at 1 and 3 months. Additionally, we will assess the incidence of major complications associated with ACLF, including hepatic encephalopathy, spontaneous bacterial peritonitis, and hepatorenal syndrome.

The ultimate goal of this investigation is to determine the relative merits of ELSS, thereby informing clinical decision‐making, optimizing patient management strategies in ACLF, and ultimately enhancing patient survival quality.

## 2 METHODS

### 2.1 Design

This systematic review and meta‐analysis was conducted to evaluate the comparative effectiveness of ELSS and standard pharmacological treatments in patients with ACLF. A comprehensive search strategy was implemented to retrieve relevant studies from the PubMed, Web of Science, EMBASE, and Google Scholar databases. The search period extended from January 1, 2000, to May 31, 2023. The primary outcomes of interest were 1‐month and 3‐month mortality rates, and the secondary outcomes included the incidence of hepatic encephalopathy, spontaneous bacterial peritonitis, and hepatorenal syndrome.

The methodological approach for the systematic review and meta‐analysis adhered to the guidelines provided in the Cochrane Handbook for Systematic Reviews of Interventions.^3^ The reporting of this review conformed to the standards of the Preferred Reporting Items for Systematic Reviews and Meta‐Analyses (PRISMA).^4^ The protocol for this study was prospectively registered with the International Prospective Register of Systematic Reviews (PROSPERO) under the registration number CRD42023428744.

### 2.2 Search strategy

This systematic review and meta‐analysis focused on designing a highly sensitive search strategy that combined free text and keyword search term synonym clusters for ACLF, free text and keyword search term synonym clusters for ELSS, free text and keyword search term synonym clusters for standard drug regimens, with term clusters for 1‐month mortality rate, 3‐month mortality rate, the incidence of hepatic encephalopathy, the incidence of spontaneous peritonitis incidence, and hepatorenal syndrome incidence word clusters. We then systematically searched for articles published in PubMed, Web of Science, EMBASE, CKNI, Preprint servers (Biorxiv (http://www.biorxiv.org), Medrxiv (http://www.biorxiv.org), and Chinaxiv (http://biotech.chinaxiv.org/)) using the keywords “acute‐on‐chronic liver failure”; “extracorporeal liver support system”; “standard drug regimen”; and “1‐month mortality, 3‐month mortality, incidence of hepatic encephalopathy, incidence of spontaneous peritonitis, and incidence of hepatorenal syndrome”. All such searches were conducted from January 1, 2000, to May 31, 2023, as described above. The reference lists of all included articles were also reviewed for potential citation eligibility.

### 2.3 Study selection and data extraction

Two reviewers independently performed a two‐step selection process, screening studies by title and abstract, followed by full‐text review. Studies were included if they were literature comparing the combined effects of clinical prognosis and complications of treatment with ELSS and standard medications, respectively, in patients with ACLF and reported on measures of survival prognostic outcome metrics (focusing on 1‐month mortality and 3‐month mortality) and the presence or absence of complications (incidence of hepatic encephalopathy, incidence of spontaneous peritonitis, and incidence of hepatorenal syndrome).

Inclusion criteria were as follows: (1) literature that would include ELSS and standard pharmacologic treatments applied to patients diagnosed with ACLF; (2) survival prognostic outcome indices that would include 1‐month mortality and 3‐month mortality; and (3) complications that would include hepatic encephalopathy, spontaneous peritonitis, and hepatorenal syndrome.

Exclusion criteria are as follows: (1) non‐human studies; (2) non‐comparative studies; (3) studies that do not include patients with ACLF; (4) studies that are not ELSS versus standard treatment regimens; (5) studies that do not have usable data to be extracted; and (6) non‐original studies (letters, reviews, and editorials).

**FIGURE 1:**
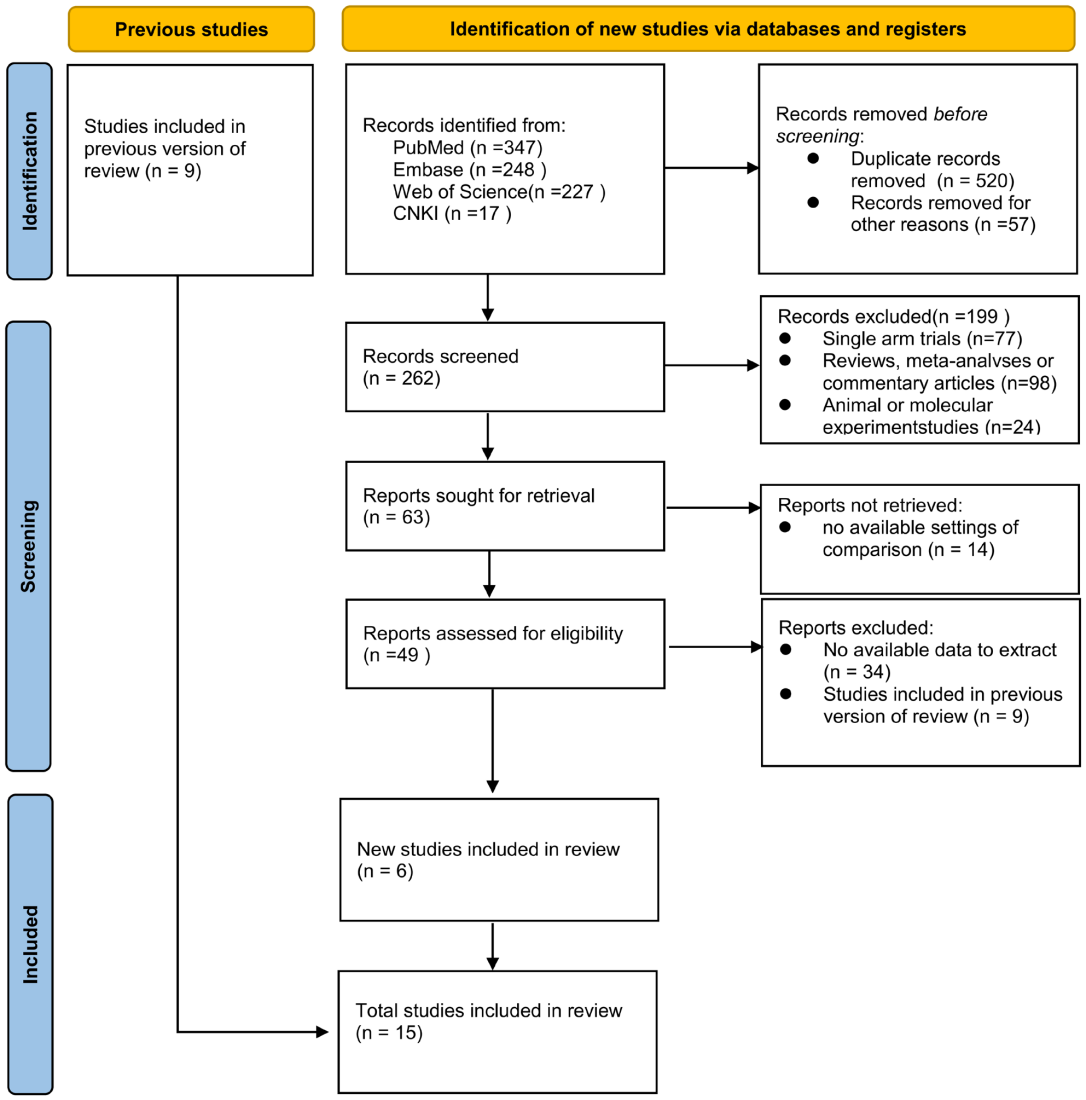
PRISMA flowchart of literature research and selection for updated meta‐analysis. [Color figure can be viewed at wileyonlinelibrary.com]

Data extraction was performed using a standardized data extraction form. Data were extracted from article text, tables, and charts. The data collected were used for study design and setting, patient age profile, gender profile, disease profile, etc. Conflicts were resolved through consensus discussions. The flowchart illustrating the literature research and selection process for the updated meta‐analysis is shown in Figure 1.

### 2.4 Statistical analysis

ORR, DCR, PFS, OS, SAEs, and other indicators are expressed as mean and standard deviations (SDs) or median and interquartile ranges (IQR) or ranges. Suppose the data for ORR, DCR, PFS, OS, SAEs, and other indicators are expressed in median IQR in the original article. In that case, the data will be transformed using methods and tools according to Professors Luo^5^ and Wan^6^ (https://www.math.hkbu.edu.hk/~tongt/papers/median2mean.html). As the data permits, clinical features (ORR, DCR, PFS, OS, and SAEs) or measurements related to downstream clinical outcomes are presented as the outputs of statistical analysis software. Statistical analysis was performed using Review Manager 5.4 (Cochrane et al., UK) and STATA 12.0 (StataCorp et al., Station, Texas). Binary variables will be compared using probability ratios (OR) with 95% confidence intervals (CIs). For continuous variable, a weighted mean difference (WMD) and a CI of 95% are calculated. According to the method described by Wan et al.,^3^ the median and quartile ranges of continuous data will be converted to mean and SD. Cochrane Qp values and *I* ^2^ statistics will be used for all meta‐analyses to check for heterogeneity. *p*‐values <0.05 or *I* ^2^ >50% denote significant heterogeneity, and a random‐effects model will be used to combine the results. Otherwise, a fixed‐effect model will be used. *p*‐value <0.05 denotes statistical significance. We will perform sensitivity tests to assess publication bias.

### 2.5 Literature quality evaluation

The quality of the included articles was assessed using the Newcastle‐Ottawa Scale (NOS),^7^ as shown in Table 1.

**TABLE 1:** NOS scale for literature quality assessment.^a^

No.	Study	Selection				Comparability	Exposure			Scores
		Adequate definition of cases	Representativeness of the cases	Selection of controls	Definition of controls	Control for important factor	Ascertainment of exposure	Same method of ascertainment for cases and controls	Non‐Responese rate	
1	Yue‐Meng 2015	★	★	★	★	★★	★	★	★	9
2	Fan 2017	★	★	★	★	★☆	★	★	★	8
3	Chen 2021	★	☆	★	★	★☆	★	★	★	7
4	Xiao 2019	★	☆	★	★	★☆	★	★	★	7
5	Sen 2004	★	★	★	★	★☆	★	★	★	8
6	Kribben 2012	★	★	★	★	★★	★	★	★	9
7	Yang 2020	★	★	★	★	★☆	★	★	★	8
8	Liu 2020	★	★	★	★	★☆	★	★	★	8
9	Wu 2023	★	★	★	★	★☆	★	★	★	8
10	Du 2005	★	★	★	★	☆☆	★	★	★	7
11	Maiwall 2021	★	★	★	★	★☆	★	★	★	8
12	Xiao 2021	★	★	★	★	★☆	★	★	☆	7
13	Mao 2010	★	☆	★	★	★☆	★	★	★	7
14	Xia 2014	★	☆	★	★	☆☆	★	★	★	6
15	Qin 2014	★	☆	★	★	★☆	★	★	☆	6

Abbreviation: NOS, Newcastle‐Ottawa Scale.

^a^ ★means 1 point, while ☆ means 0 point.

## 3 RESULTS

### 3.1 Search results

We obtained a total of 521 articles from online databases and other sources, of which 7 were eliminated as duplicates. Based on titles and abstracts, 472 articles were removed from them. A total of 49 published papers were considered for full‐text assessment, and 15 studies were included in the final quantitative analysis,^8^, ^9^, ^10^, ^11^, ^12^, ^13^, ^14^, ^15^, ^16^, ^17^, ^18^, ^19^, ^20^, ^21^, ^22^ as shown in Table 2.

**TABLE 2:** Table of basic information about the included articles.

No.	Study	Setting	Groups setting	Design	No of participants	Age, y (median, IQR or mean ± SD)	Male, *n* %	MELD score (median, IQR or mean ± SD)	Tbil (median, IQR or mean ± SD)
1	Yue‐Meng 2015	A hospital in China	ELSS group vs. SMT group	Retrospective cohort, single center, Non‐RCT	158 ACLF patients	ELSS group: 51.4 ± 5.6 SMT group: 52.1 ± 6.6	ELSS group: 71.1% SMT group: 85.0%	ELSS group: 24.3 ± 1.3 SMT group: 22.3 ± 1.2	ELSS group: 19.8 ± 3.37 mg/dL SMT group: 20.9 ± 3.26 mg/dL
2	Fan 2017	A hospital in China	ELSS group vs. SMT group	Retrospective cohort, single center, non‐RCT	560 ACLF patients	ELSS group: 42.1 ± 11.5 SMT group: 49.2 ± 13.2	ELSS group: 92.3% SMT group: 83.3%	ELSS group: 24.9 (21.9–28.9) SMT group: 25.0 (20.0–30.0)	ELSS group: 22.4 (15.9–28.1) mg/dL SMT group: 19.6 (13.1–27.0) mg/dL
3	Chen 2021	15 hospital in China	ELSS group vs. SMT group	Prospective cohort, multicenter, RCT	524 ACLF patients	ELSS group: 47.47 ± 11.40 SMT group: 48.31 ± 11.17	ELSS group: 87.35% SMT group: 82.40%	ELSS group: 31.73 ± 5.37 SMT group: 31.12 ± 5.60	ELSS group: 437.48 ± 162.20 μmol/L SMT group: 380.17 ± 188.88 μmol/L
4	Xiao 2019	11 hospital in China	ELSS group vs. SMT group	Retrospective cohort, multicenter, non‐RCT	790 ACLF patients	ELSS group: 44.0 ± 10.6 SMT group: 46.9 ± 11.4	ELSS group: 85.2% SMT group: 86.0%	ELSS group: 25.9 ± 5.2 SMT group: 24.8 ± 5.6	ELSS group: 22.1 ± 7.4 mg/dL SMT group: 22.4 ± 7.0 mg/dL
5	Sen 2004	A hospital in England	ELSS group vs. SMT group	Prospective cohort, single center, RCT	18 ACLF patients	ELSS group: 45.0 (34.0–52.0) SMT group: 44.0 (33.0–64.0)	ELSS group: 77.8% SMT group: 66.6%	ELSS group: 16.5 (13.1–31.2) SMT group: 19.4 (4.3–25.2)	ELSS group: 396.0 (281.0–708.0) μmol/L SMT group: 232.0 (115.0–416.0) μmol/L
6	Kribben 2012	10 hospital in Europe	ELSS group vs. SMT group	Prospective cohort, multicenter, RCT	145 ACLF patients	ELSS group: 50.0 ± 11.0 SMT group: 51.0 ± 9.0	ELSS group: 62.3% SMT group: 64.7%	ELSS group: 28.0 ± 10.0 SMT group: 27.0 ± 10.0	ELSS group: 26.0 ± 15.0 mg/dL SMT group: 25.0 ± 14.0 mg/dL
7	Yang 2020	13 hospital in China	ELSS group vs. SMT group	Prospective cohort, multicenter, non‐RCT	924 ACLF patients	ELSS group: 44.0 ± 17.0 SMT group: 47.0 ± 16.0	ELSS group: 89.5% SMT group: 87.5%	NA	ELSS group: 24.3 ± 9.4 mg/dL SMT group: 19.7 ± 10.4 mg/dL
8	Liu 2020	A hospital in China	ELSS group vs. SMT group	Retrospective cohort, single center, non‐RCT	425 ACLF patients	ELSS group: 48.27 ± 11.93 SMT group: 50.61 ± 12.57	ELSS group: 80.77% SMT group: 79.63%	ELSS group: 23.21 ± 6.44 SMT group: 23.87 ± 8.22	ELSS group: 308.55 (240.6–387.7) μmol/L SMT group: 308.90 (262.9–413.7) μmol/L
9	Wu 2023	A hospital in China	ELSS group vs. SMT group	Prospective cohort, single center RCT	186 ACLF patients	ELSS group: 48.23 ± 11.44 SMT group: 49.06 ± 13.72	ELSS group: 90.32% SMT group: 87.10%	ELSS group: 21.33 ± 3.28 SMT group: 19.93 ± 4.55	ELSS group: 413.59 ± 174.23 μmol/L SMT group: 397.39 ± 154.16 μmol/L
10	Du 2005	A hospital in China	ELSS group vs. SMT group	Prospective cohort, single center, non‐RCT	650 ACLF patients	ELSS group: 40.4 ± 10.5 SMT group: 38.1 ± 12.4	ELSS group: 86.1% SMT group: 85.9%	NA	ELSS group: 609.82 ± 196.95 mmol/L SMT group: 586.67 ± 212.81 mmol/L
11	Maiwall 2021	AARC database	ELSS group vs. SMT group	Retrospective cohort, multicenter, non‐RCT	208 ACLF patients	ELSS group: 45.5 ± 13.4 SMT group: 42.7 ± 11.1	ELSS group: 89.9% SMT group: 92.1%	ELSS group: 26.0 ± 7.8 SMT group: 27.0 ± 7.4	NA
12	Xiao 2021	10 hospital in China	ELSS group vs. SMT group	Retrospective cohort, multicenter, non‐RCT	707 ACLF patients	ELSS group: 44.0 ± 10.5 SMT group: 43.0 ± 11.4	ELSS group: 89.1% SMT group: 81.0%	ELSS group: 25.6 (22.7–28.6) SMT group: 23.9 (21.5–27.6)	ELSS group: 378.6 (271.1–514.9) μmol/L SMT group: 296.8 (202.5–423.3) μmol/L
13	Mao 2010	A hospital in China	ELSS group vs. SMT group	Retrospective cohort, single center, non‐RCT	193 ACLF patients	ELSS group: 48.3 ± 12.5 SMT group: 43.7 ± 10.9	ELSS group: 87.1% SMT group: 86.1%	NA	ELSS group: 617.3 ± 184.3 mmol/L SMT group: 591.6 ± 228.3 mmol/L
14	Xia 2014	A hospital in China	ELSS group vs. SMT group	Retrospective cohort, single center, non‐RCT	787 ACLF patients	ELSS group: 45.0 (28.0–562.0) SMT group: 46.0 (28.0–64.0)	ELSS group: 85.5% SMT group: 80.1%	ELSS group: 25.0 (18.0–32.0) SMT group: 25.0 (15.0–35.0)	ELSS group: 33.3 ± 3.70 mg/dL SMT group: 32.4 ± 4.80 mg/dL
15	Qin 2014	A hospital in China	ELSS group vs. SMT group	Prospective cohort, single center, RCT	234 ACLF patients	ELSS group: 44.13 ± 17.03 SMT group: 48.66 ± 18.55	ELSS group: 82.69% SMT group: 72.31%	ELSS group: 28.56 ± 4.52 SMT group: 29.46 ± 6.03	ELSS group: 17.95 ± 8.25 mg/dL SMT group: 16.12 ± 8.54 mg/dL

Abbreviations: ACLF, acute‐on‐chronic liver failure; ELSS, extracorporeal liver support systems; IQR, interquartile range; MELD, model for end‐stage liver disease; NA, not available; RCT, randomized controlled trial; SD, standard deviation; SMT, standard medical treatment; Tbil, total bilirubin.

### 3.2 Forests plots

**FIGURE 2:**
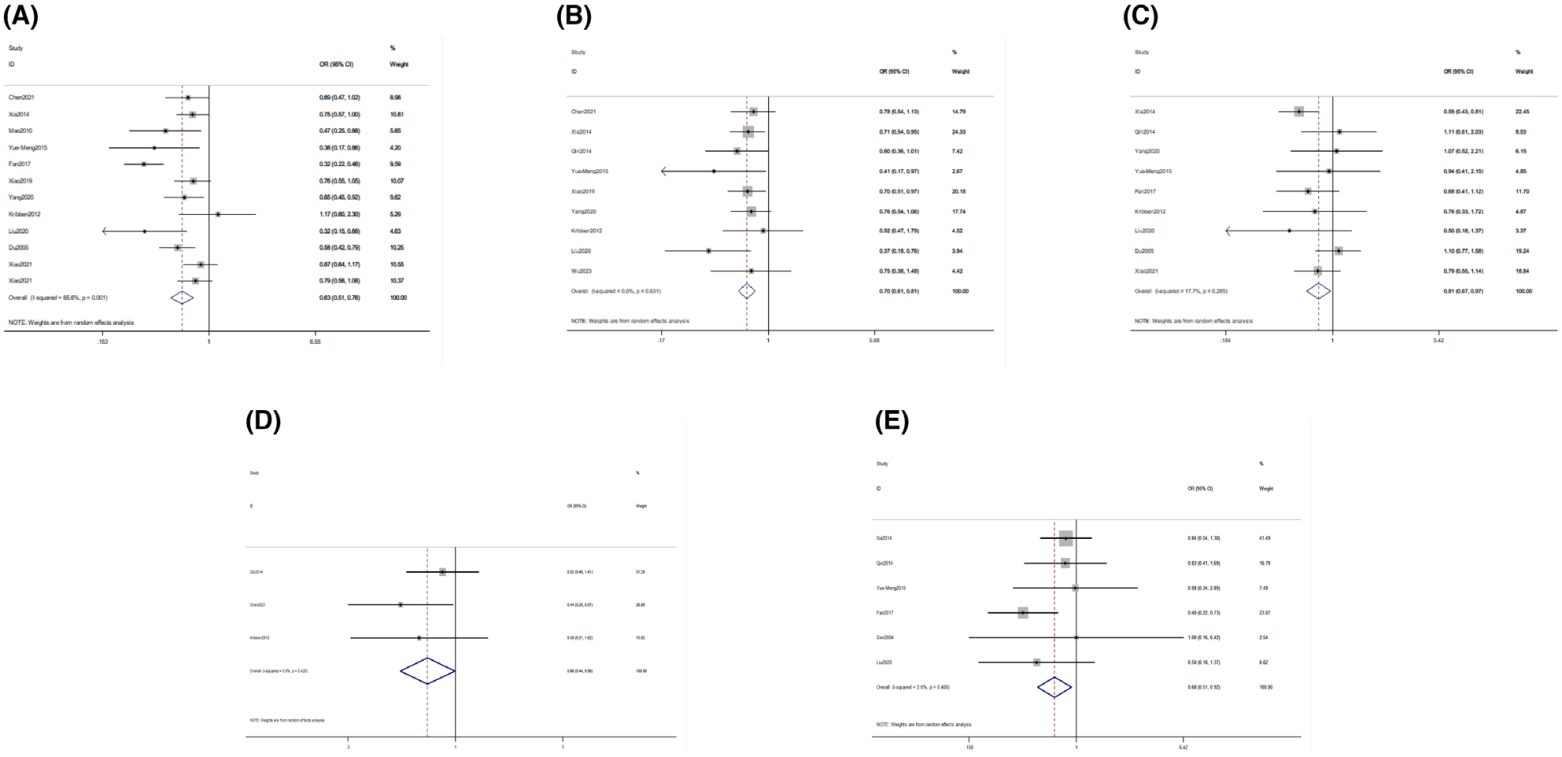
Forest plot of comparing artificial liver support system therapy and standard drug therapy (A: 1‐month mortality, B: 3‐month mortality, C: hepatic encephalopathy, D: spontaneous peritonitis, and E: hepatorenal syndrome). [Color figure can be viewed at wileyonlinelibrary.com]

A total of 6502 patients from 15 studies were included in this meta‐analysis; we produced forest plots of the relevant studies, as shown in Figure 2.

Since some of the forest plot results of the included studies showed that *I* ^2^ was greater than 50%, we further produced funnel plots and sensitivity analyses of the relevant studies, as shown in Figures 3 and 4.

### 3.3 Funnel plots

**FIGURE 3:**
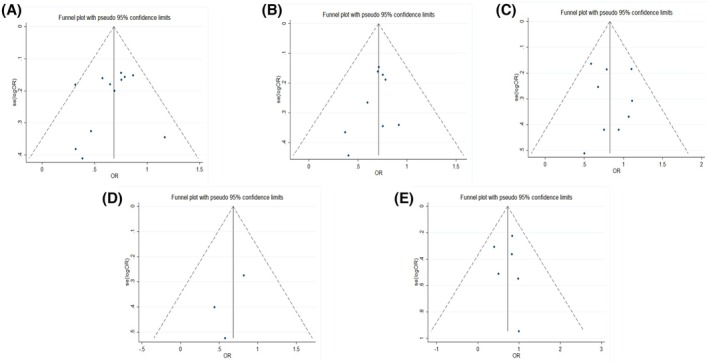
Funnel plots of comparing artificial liver support system therapy and standard drug therapy (A: 1‐month mortality, B: 3‐month mortality, C: hepatic encephalopathy, D: spontaneous peritonitis, and E: hepatorenal syndrome). [Color figure can be viewed at wileyonlinelibrary.com]

### 3.4 Sensitivity analysis

**FIGURE 4:**
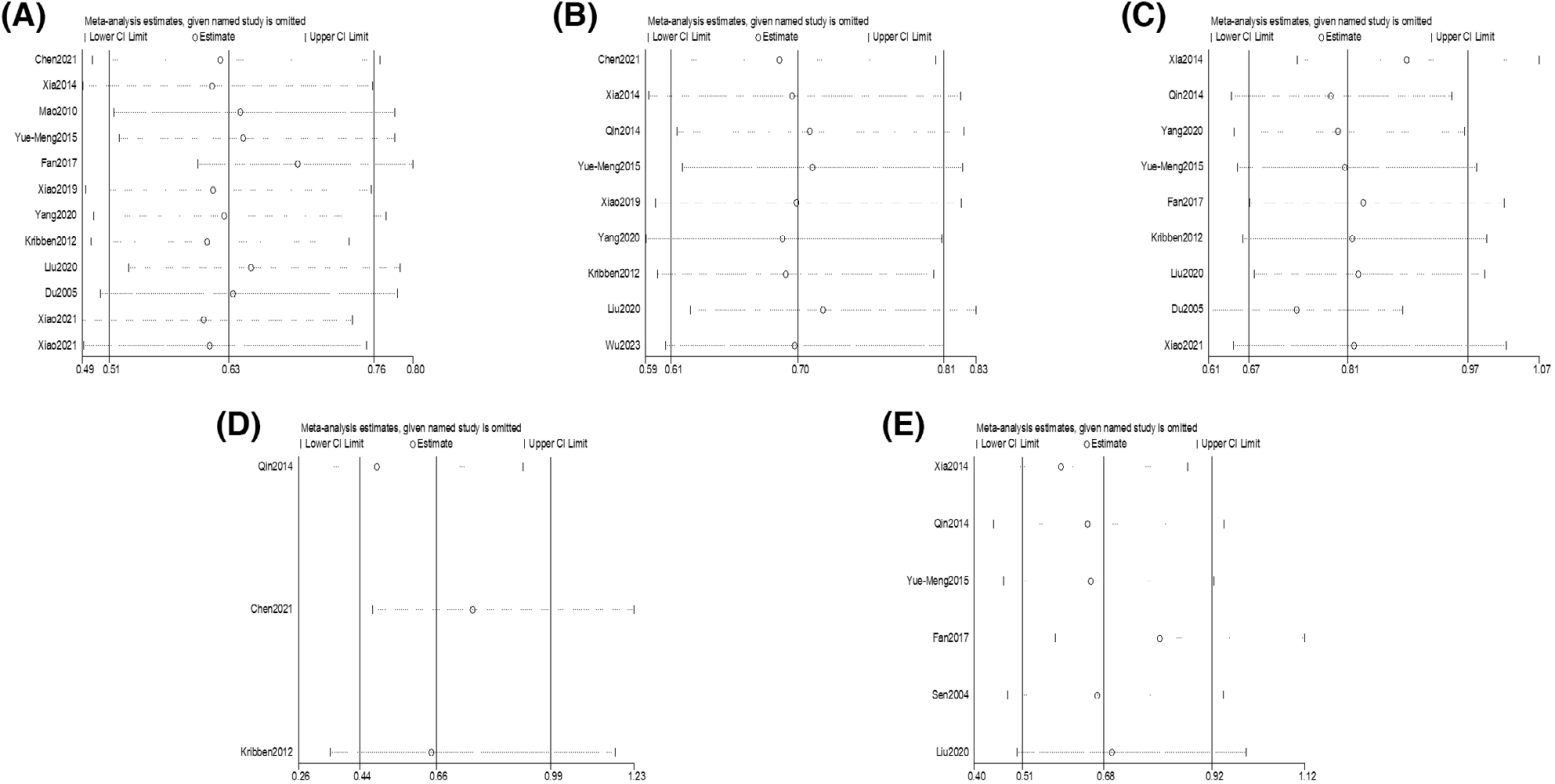
Sensitivity analysis of comparing artificial liver support system therapy and standard drug therapy (A: 1‐month mortality, B: 3‐month mortality, C: hepatic encephalopathy, D: spontaneous peritonitis, and E: hepatorenal syndrome).

### 3.5 Subgroup analysis

Based on the above analysis, we also further analyzed the subgroup analysis of age, gender, MELD score, and Tbil concentration in the original paper reporting the combined effect of extracorporeal support system and standard drug therapy on the 1‐month mortality, 3‐month mortality, incidence of hepatic encephalopathy, incidence of spontaneous peritonitis, and incidence of hepatorenal syndrome in patients with slow‐accelerated hepatic failure, and the results are shown in Table 3.

**TABLE 3:** Subgroup and sensitivity analyses for the primary outcomes.

	No. of stud OR	95% Cl	*p*	*I* ^2^ (%)
Extracorporeal liver support systems (ELSS) vs standard medical treatment				
1 months mortality				
Age				
≥45	9	0.69 (0.58, 0.81)	0.189	28.8
<45	3	0.55 (0.31, 0.95)	0.0000	88.8
Male%				
≥75%	9	0.60 (0.48, 0.76)	0.001	69.5
<75%	3	0.73 (0.45, 1.18)	0.116	53.5
Tbil				
≥23 mg/dL	6	0.70 (0.59, 0.82)	0.332	12.9
<23 mg/dL	6	0.54 (0.37, 0.79)	0.0001	80.0
3 months mortality				
Male%				
≥75%	6	0.71 (0.61, 0.83)	0.609	0.0
<75%	3	0.64 (0.43, 0.95)	0.325	11.1
MELD score				
≥24	7	0.72 (0.62, 0.83)	0.813	0.0
<24	2	0.53 (0.27, 1.07)	0.158	49.9
Tbil				
≥23 mg/dL	5	0.74 (0.62, 0.88)	0.958	0.0
<23 mg/dL	4	0.58 (0.42, 0.81)	0.237	29.2
Hepatic encephalopathy				
Age				
≥45	5	0.67 (0.52, 0.86)	0.510	0.0
<45	4	0.90 (0.72, 1.13)	0.336	11.3
Male%				
≥75%	6	0.89 (0.73, 1.09)	0.429	0.0
<75%	3	0.64 (0.48, 0.85)	0.531	0.0
MELD score				
≥24	7	0.83 (0.67, 1.03)	0.181	32.3
<24	2	0.64 (0.34, 1.21)	0.538	0.0
Tbil				
≥23 mg/dL	3	0.79 (0.50, 1.26)	0.039	69.3
<23 mg/dL	6	0.82 (0.65, 1.04)	0.685	0.0
Hepatorenal syndrome				
Age				
≥45	4	0.80 (0.55, 1.16)	0.787	0.0
<45	2	0.56 (0.27, 1.15)	0.123	57.9
Male%				
≥75%	4	0.55 (0.36, 0.83)	0.422	0.0
<75%	2	0.86 (0.57, 1.29)	0.782	0.0
MELD score				
≥24	4	0.69 (0.46, 1.04)	0.204	34.7
<24	2	0.59 (0.24, 1.42)	0.524	0.0
Tbil				
≥23 mg/dL	2	0.84 (0.55, 1.30)	0.854	0.0
<23 mg/dL	4	0.59 (0.38, 0.90)	0.324	13.6

Abbreviations: MELD, model for end‐stage liver disease; Tbil, total bilirubin.

Overall, this meta‐analysis conducted a funnel plot analysis to assess publication bias, and the results suggested that there is no publication bias in this meta‐analysis. Additionally, sensitivity and subgroup analyses were performed, with the sensitivity analysis indicating that the sources of heterogeneity may be age, gender ratio, MELD score, and total bilirubin levels in patients.

## 4 DISCUSSION

ACLF delineates a critical syndrome where patients with previously stable chronic liver disease precipitously decompensate due to various acute insults. The definitional boundaries of ACLF, however, remain a subject of considerable debate. The European Association for the Study of the Liver (EASL) in 2013 discerned ACLF from mere acute decompensation of cirrhosis, describing it as an intricate syndrome of acute deterioration superimposed on chronic cirrhosis—irrespective of its compensated or decompensated nature—manifesting with multiorgan failure, including that of extrahepatic systems, and concomitant with elevated short‐term morbidity and mortality rates (≥15% within 28 days).^23^ The North American Consortium for the Study of End‐Stage Liver Disease (NACSELD) further refined the criteria in 2014, stipulating the disease's diagnosis with the coexistence of at least two severe extrahepatic organ failures, categorically including shock, hepatic encephalopathy of grade 3 or 4, the necessity for renal replacement therapy, or mechanical ventilation.^24^ The Chinese guidelines for liver failure in 2018 characterized ACLF in the milieu of chronic liver disease through a spectrum of precipitants, leading to an acute exacerbation of jaundice (Tbil ≥10 times the upper limit of normal or an incremental daily rise ≥17.1 μmol/L) and coagulopathy (PTA ≤40% or INR ≥1.5), potentially accompanied by a suite of complications such as hepatic encephalopathy, ascites, electrolyte imbalances, infections, hepatorenal syndrome, hepatopulmonary syndrome, and extrahepatic organ failure. The guidelines further stratified ACLF into three distinct categories based on the underlying chronic liver condition: type A (chronic non‐cirrhotic liver disease), type B (compensated cirrhosis), and type C (decompensated cirrhosis).^21^ In 2019, the consensus from the Asia‐Pacific Association for the Study of Liver (APASL) defined ACLF as an acute hepatic insult manifesting initially as jaundice (Tbil ≥5 mg/dL) and/or coagulation dysfunction (INR ≥1.5 or PTA <40%) in a patient with known or undiagnosed chronic liver disease, evolving to include ascites and/or hepatic encephalopathy within four weeks of onset and tied to a significant 28‐day morbidity and mortality.^25^ More recently, in 2022, the American College of Gastroenterology (ACG) described ACLF as a potentially reversible condition that in the absence of intervention—via treatment of the underlying liver ailment, hepatic supportive care, or liver transplantation—could culminate in multiorgan failure and patient demise within three months. The ACG underscored the recognition of ACLF through chronic liver disease markers, elevated Tbil, and a prolonged INR, with the advent of renal, respiratory, circulatory, or cerebral failures as corroborative of the diagnosis.^26^ Accurate identification and management of this complex syndrome are imperative and should be prioritized in clinical settings.

The pathophysiology of ACLF is intricately linked to a robust systemic inflammatory response, which is initiated by pathogen‐associated molecular patterns (PAMPs) and damage‐associated molecular patterns (DAMPs) in the circulation.^27^ Clària et al., in their seminal 2016 study, elucidated that the severity of systemic inflammation in ACLF patients was directly proportional to the incidence of organ failure and short‐term mortality.^28^ The cascade leading to organ failure in ACLF is likely multifactorial, involving a synergy of suboptimal tissue perfusion, immune‐mediated damage, and mitochondrial dysfunction. Fernández et al., in 2018, highlighted bacterial infections as pivotal precipitants of systemic inflammation in ACLF, capable of inciting profound inflammatory responses, consequent complications, and elevated mortality rates.^29^

Furthermore, ACLF represents a frequent and severe complication among patients with alcoholic hepatitis (AH). A pivotal finding by Moreau et al. in 2013 indicated that 60% of individuals with ACLF had concomitant AH.^23^ The pathogenic bridge between chronic alcohol consumption and ACLF is characterized by a progression from hepatic steatosis to inflammation, fibrogenesis, and eventually, severe AH. This trajectory fosters immune dysregulation, augmenting susceptibility to infections and propelling the system toward a hyperinflammatory state. Such an exacerbated inflammatory milieu is a precursor to the systemic organ failure that typifies ACLF.^30^

ACLF presents as a multifaceted syndrome characterized by swift clinical deterioration, high short‐term mortality, and a spectrum of clinical manifestations. Patients with ACLF may experience a range of complications including hepatic encephalopathy, spontaneous bacterial peritonitis, hepatorenal syndrome, circulatory shock, severe infections, gastrointestinal hemorrhage, and electrolyte imbalances. The heterogeneity of ACLF phenotypes, coupled with gaps in understanding its pathogenesis, often leads to diagnostic and therapeutic delays, complicating the implementation of early and standardized interventions.

Recent advancements have begun to clarify therapeutic strategies tailored to specific etiologies of ACLF. For instance, nucleoside analog antivirals have shown efficacy in hepatitis B‐related ACLF, glucocorticoids in autoimmune hepatitis, and N‐acetylcysteine in cases of acetaminophen toxicity.^31^ Liver transplantation remains the definitive treatment for end‐stage liver disease, providing a life‐saving option for eligible patients.

Concurrently, ELSS, encompassing non‐biological and bioartificial devices, have emerged as supplemental therapeutic modalities. These systems transiently substitute for hepatic functions—detoxification, synthesis, and regulation—thereby supporting hepatocyte regeneration in patients with self‐limited liver insults or bridging to transplantation for those awaiting a donor organ. The integration of such systems into comprehensive management protocols holds promise for sustaining vital functions, minimizing transplantation‐related complications, and ultimately, improving patient outcomes.^32^

ELSS, encompassing non‐biological ALSS, provide a crucial therapeutic avenue for patients afflicted with liver failure. The cornerstone of ELSS is to transiently supplant hepatic functions through mechanical, chemical, or other non‐biological means, thereby bridging the gap until spontaneous liver regeneration or liver transplantation becomes feasible. The rationale behind this approach leverages the liver's inherent capacity for repair and its potent regenerative potential. By mitigating hepatic insufficiency and optimizing the internal milieu, ELSS aims to facilitate the recovery of liver function or to serve as a preparatory step prior to definitive therapies.

Contemporary ELSS modalities include the molecular adsorbent recirculation system (MARS), single‐pass albumin dialysis (SPAD), Prometheus system, selective plasma filtration therapy, and hemodialysis filtration. These systems, augmented by advances in materials science, cell engineering, and clinical medicine, have become integral in managing liver failure cases.^32^

Despite the technological advancements, the efficacy of ELSS in ACLF remains a subject of debate. In 2003, Kjaergard et al. suggested that ELSS could reduce mortality rates relative to conventional pharmacotherapy, albeit with a heightened risk of serious complications, including hemorrhage and infection.^33^ Subsequent studies yielded mixed outcomes; for example, Liu et al. in 2004 reported a 33% mortality reduction in ACLF patients utilizing ELSS, which was, however, not devoid of potential complications.^34^ In contrast, a study by Zheng et al. in 2013 indicated a significant mortality decrease without an increased incidence of hepatic encephalopathy or bleeding.^35^ More recently, a 2020 study by Liu et al. identified ELSS as an independent predictor of improved 28‐ and 90‐day survival rates in patients with HBV‐ACLF, with combination therapy (drugs plus ELSS) outperforming standard pharmacotherapy as per the COSSH‐ACLF benchmark.^18^

Our meta‐analysis has provided compelling statistical evidence supporting the efficacy of ELSS in patients with ACLF. The data demonstrate significant reductions in mortality and complications when compared to standard pharmacotherapy. Specifically, the odds ratio (OR) for 1‐month mortality was 0.63 (95% CIs: 0.51, 0.76), with moderate heterogeneity (I^2 = 65.6%, *p* = 0.001). For 3‐month mortality, the OR was 0.70 (95% CI: 0.61, 0.81), with no observed heterogeneity (I^2 = 0.0%, *p* = 0.631). Compared to the previously published meta‐analysis on the application of artificial liver technology in ACLF,^34^, ^35^ which included a smaller number of studies, this meta‐analysis incorporates a greater number of articles (*n* = 15). Additionally, this meta‐analysis employed robust statistical analyses, such as funnel plots, to assess publication bias, sensitivity analysis, and subgroup analysis to investigate sources of heterogeneity in the meta‐analysis. Furthermore, this meta‐analysis evaluated a greater number of outcome measures and complications compared to the earlier meta‐analysis. Therefore, the comprehensive results of this meta‐analysis are more representative and reliable.

Additionally, ELSS was associated with a lower odds of developing hepatic encephalopathy (OR = 0.81, 95% CI: 0.67, 0.97, I^2 = 17.7%, *p* = 0.285), spontaneous peritonitis (OR = 0.66, 95% CI: 0.44, 0.99, I^2 = 0.0%, *p* = 0.420), and hepatorenal syndrome (OR = 0.68, 95% CI: 0.51, 0.92, I^2 = 2.6%, *p* = 0.400), indicating a beneficial effect in managing these complications.

Subgroup analysis further revealed that the therapeutic advantages of ELSS extend across various age groups in the ACLF patient population. Notably, ELSS appears to confer a greater therapeutic benefit in patients with lower model for end‐stage liver disease (MELD) scores and lower total bilirubin (Tbil) concentrations. This suggests that ELSS could be particularly advantageous for patients with less severe liver dysfunction as indicated by these parameters.

In conclusion, our findings advocate for the integration of ELSS into the treatment paradigm for ACLF, highlighting its potential to improve survival rates and reduce the incidence of critical complications, with particular benefit in specific patient subpopulations.

ACLF presents as a rapidly progressing disease characterized by high short‐term mortality and a spectrum of clinical phenotypes. A multicenter prospective study by Verma et al., grounded in the Asia‐Pacific Association for the Study of the Liver Expert Consensus (APASL‐ACLF) definition, reported a 30‐day survival rate of 64.9% among 2864 patients in 2021.^36^ In the following year, Mezzano et al. conducted a meta‐analysis to evaluate the global burden of ACLF over the past decade, utilizing the European Association for the Study of the Liver's definition. They reported a staggering 90‐day mortality rate of 58%, identifying alcohol as the predominant etiological factor, infections as the primary precipitating events, and renal dysfunction as the most frequent organ failure.^37^

These findings underscore the grave prognosis associated with ACLF and the urgent need for effective therapeutic interventions. Our meta‐analysis corroborates the notion that the use of ELSS can result in lower 1‐month and 3‐month mortality rates compared to standard pharmacotherapy in patients with hepatorenal syndrome. Moreover, ELSS has been shown to significantly enhance survival outcomes in liver failure patients. The underlying therapeutic mechanism exploits the remarkable regenerative capacity of hepatocytes. By employing extracorporeal mechanical, physicochemical, and biological devices, ELSS facilitates the removal of toxins, replenishment of vital substances, and amelioration of the internal milieu. This approach not only serves to temporarily assume some functions of the failing liver but also fosters an environment conducive to hepatocyte regeneration and hepatic restoration or, alternatively, bridges the gap to liver transplantation.

Hepatic encephalopathy (HE) represents a spectrum of neuropsychiatric anomalies resulting from advanced hepatic insufficiency and/or portosystemic shunting. Clinical manifestations of HE vary widely, extending from subtle cognitive impairments to deep coma. The pathogenesis of HE is multifaceted, primarily involving the diversion of ammonia and other neurotoxins away from hepatic metabolism via portosystemic collaterals, culminating in cerebral exposure to these deleterious substances.

Yoshiba et al.'s seminal 1993 study marked the initial observation that ELSS could mitigate the symptoms of hepatic coma in ACLF patients, providing a pivotal insight into the management of HE.^38^ Subsequently, a 2019 investigation by Huang et al. reinforced the significance of HE as a determinant of outcomes in ACLF patients undergoing artificial liver treatment.^39^

Building on these foundational studies, our meta‐analysis has yielded further evidence that the incidence of HE is significantly reduced in ACLF patients treated with ELSS compared to those receiving conventional pharmacotherapy. By assuming critical liver functions, the ELSS alleviates the neuropsychiatric disorders associated with liver failure, enhances metabolic and detoxification processes, and facilitates the systemic removal of accumulated toxins. The system's capacity to replenish essential coagulation factors and albumin also contributes to the stabilization of the patient's condition and supports hepatic recovery.

In light of these findings, the application of ELSS demonstrates clear clinical benefits in the management of HE within the ACLF patient cohort, underscoring its role in improving patient outcomes and offering a bridge to liver function recovery or transplantation.

Bacterial infections are pivotal in the pathogenesis and exacerbation of ACLF, often serving as both an inciting event and a complication of the syndrome. Predominantly, Gram‐positive bacteria are implicated in triggering ACLF, although Gram‐negative bacteria also play a significant role. Spontaneous bacterial peritonitis (SBP) is notably recognized for its dual role in precipitating and complicating ACLF.^40^

In 2018, research by Fernández et al. highlighted the prevalence of bacterial infections in precipitating ACLF, noting that the syndrome itself predisposes patients to further infections, which are associated with severe systemic inflammation, worsened clinical morbidity, and elevated mortality rates.^29^ Jacques et al., in 2021, reinforced this notion, demonstrating a clear association between SBP and decreased survival in ACLF patients.^41^ Most recently, Liu et al. (2023) identified interleukin‐10 (IL‐10) as an independent prognostic marker in patients with concomitant SBP and ACLF.^42^

The present meta‐analysis ventures into a less frequently reported domain, assessing the impact of ELSS on morbidity in ACLF patients, particularly those with SBP. Our findings corroborate a reduced morbidity in ACLF patients managed with ELSS as compared to those treated with standard therapies for SBP. Given the grim prognosis associated with delayed infection management in ACLF, it is imperative to consider a concurrent underlying infection in these patients and to initiate comprehensive sepsis screening promptly.

Immediate commencement of antibiotic therapy based on preliminary testing is recommended, without awaiting microbiological culture confirmation. Third‐generation cephalosporins are advocated as the initial agents for community‐acquired infections. A critical reassessment of patients’ response to treatment is warranted 48 hours post‐initiation to refine the therapeutic approach accordingly. Moreover, in patients with spontaneous hepatorenal syndrome, albumin replacement therapy is advised to preempt SBP and enhance prognosis. For high‐risk patients, prophylactic antimicrobial therapy is warranted to prevent potential infections.

Hepatorenal syndrome (HRS) is characterized as a form of functional renal failure, manifesting as progressive prerenal azotemia in patients with advanced liver disease. It is particularly prevalent among individuals with severe cirrhosis and ascites and stands as a frequent complication in the context of ACLF.^43^

Qin et al. (2014) identified HRS as an independent prognostic indicator for elevated short‐term mortality in ACLF patients.^10^ This finding underscores the critical impact of renal function on the survival outcomes in this patient population. Further research by Sheng et al. (2022) revealed that ELSS exert a considerable beneficial influence on clinical symptomatology in HRS, with statistical significance (*p* = 0.025).^44^

The comprehensive meta‐analysis conducted as part of our investigation lends additional support to the notion that the utilization of ELSS in ACLF patients is associated with a decreased incidence of HRS when compared to standard pharmacotherapy. Furthermore, ELSS application was found to effectively enhance hepatic and renal functions and to maintain electrolyte homeostasis. These findings advocate for the vigilant monitoring of renal function dynamics in ACLF patients. Clinicians should be acutely aware of the necessity for early detection and timely intervention in the management of disease risk, which may substantially contribute to improved clinical prognoses.

This study is subject to several limitations that warrant consideration. First, the intricate and multifactorial nature of ACLF presents inherent challenges in its diagnosis and treatment, potentially leading to delays that could compromise the effectiveness of therapeutic interventions. The complexity of ACLF requires a nuanced understanding that is not yet fully developed within the medical community, thus influencing the timeliness and accuracy of clinical responses.

Second, the meta‐analysis is constrained by the limited volume of studies available for inclusion, especially since the number of randomized clinical trials included is relatively small, with only 5 studies, which inherently restricts the scope of our findings. Studies with a focus on long‐term survival and multiple complications were given precedence due to their scarcity, highlighting an acute need for larger and more extensive longitudinal research. Such studies are essential to corroborate the preliminary insights offered here and to fortify the evidence base for clinical practice in the context of ACLF. Larger, prospective, multicenter randomized controlled studies are needed for further investigation.

Finally, the broad timespan encompassing the studies included in this meta‐analysis reflects a period of evolution in the types and modalities of ELSS. This temporal variability implies that our analysis spans different generations of technology and practice patterns. Consequently, detailed investigations into the outcomes of ACLF patients managed with specific ELSS protocols are indispensable. Future research in this domain is likely to provide valuable clinical guidance and refine therapeutic strategies for managing this complex syndrome.

## 5 CONCLUSION

Our study elucidates the impact of ELSS on the clinical outcomes of patients with ACLF. The findings suggest that the implementation of ELSS in ACLF management yields enhanced survival rates and a reduction in adverse complications when juxtaposed with conventional pharmacotherapy. Notably, patients presenting with lower MELD scores and reduced total bilirubin (Tbil) levels appear to derive the most pronounced benefit from ELSS therapies.

It is crucial to interpret these results as preliminary. A more profound and comprehensive understanding of the efficacy and safety of ELSS in treating ACLF requires the assimilation of additional data from systematic, controlled, and interventional studies with robust designs. Our research team advocates for a tailored approach to ACLF treatment, emphasizing the need for customization of ELSS interventions to align with each patient's unique clinical parameters.

Prompt, standardized treatment interventions have the potential to substantially improve patient outcomes and quality of life. As such, the advancement of research into various ELSS protocols for ACLF patients holds great clinical promise. Such studies will be instrumental in refining treatment paradigms and optimizing patient management strategies in this challenging clinical context.

## AUTHOR CONTRIBUTIONS

Haiyu Liu, Zhibo Yang, Qiong Luo, and Jianhui Lin: Conceptualization, Performed the experiments, Methodology, Investigation, Data curation, Writing – original draft, Finalized manuscript, Performed the experiments, Formal analysis, Performed the experiments, Validation, Supervision, Conceptualization, Supervision, Funding acquisition, Project administration, Writing – review & editing.

## FUNDING INFORMATION

This study was supported by grants from Fujian Province Natural Science Foundation Project (Project No. 2021J011293); Beijing Liver and Gallbladder Mutual‐Aid Public Welfare Foundation Artificial Liver Special Fund (Project No. RGGJJ‐2021‐006), and the Science and Technology Planning Project of Fuzhou (No. 2022‐S‐010).

## CONFLICT OF INTEREST STATEMENT

There are no declared conflicts of interest.

## ETHICS STATEMENT

Approval of the research protocol: N/A. Informed Consent: N/A. Registry and the Registration No. of the study/trial: N/A. Animal Studies: N/A. Research involving recombinant DNA: N/A.

## DECLARATIONS RESEARCH INVOLVING HUMAN PARTICIPANTS AND/OR ANIMALS

This study did not involve the usage of animals or humans. Since this study did not involve the usage of humans, there was no need for informed consent.

## THE SYSTEMATIC REVIEW REGISTER

The study protocol has been registered on the International Prospective Register of Systematic Reviews (PROSPERO) (CRD42023428744).

## Data Availability

All data generated or analyzed during this study are included in this published article. The data that support the findings of this study are available from the corresponding author upon reasonable request.
